# Elevated serotonin alters whole-blood expression of serotonin receptor and metabolism genes in the lactating dairy cow

**DOI:** 10.3168/jdsc.2021-0108

**Published:** 2021-06-19

**Authors:** M.K. Connelly, L.L. Hernandez

**Affiliations:** Department of Animal and Dairy Sciences, University of Wisconsin, Madison 53706

## Abstract

Serotonin's role as a hormone has been demonstrated through modulating calcium metabolism, energy homeostasis, and immune function in the preweaned and lactating bovine. Recently, manipulation of the serotonergic axis in calves via administration of 5-hydroxy-l-tryptophan (5-HTP), the serotonin precursor, elicited immunomodulatory effects and regulated serotonin transport and metabolism genes in leukocytes. However, serotonin's ability to modulate whole-blood gene expression in lactating dairy cows is unknown. Our objective was to explore the effect of infusing 5-HTP on whole-blood expression of genes regulating serotonin transport (reuptake transporter), signaling (receptors), metabolism (synthesis and degradation), and cytokines in dairy cows. Twelve lactating multiparous Holstein dairy cows were blocked by parity, milk production, and days in milk in a randomized complete block design and randomly assigned to intravenous infusion of either 1 L of 1.5 mg/kg 5-HTP dissolved in saline (n = 6) or 1 L of saline solution (n = 6) for 3 consecutive days. Whole blood was collected at 48, 56, and 72 h relative to termination of first infusion to analyze whole-blood gene expression. Infusion of 5-HTP increased whole-blood mRNA expression of monoamine oxidase-A and serotonin receptor 7 across the experimental period. Forty-eight hours from termination of first infusion, the mRNA of monoamine oxidase-A, serotonin reuptake transporter, and serotonin receptor 7 were increased in blood relative to the control. Interleukin-8 concentrations and mRNA were unchanged in response to 5-HTP infusion. Collectively, these data suggest that infusion of 5-HTP may alter mRNA of serotonin metabolism, transport, and signaling genes in whole blood of lactating dairy cows.

## Highlights

• Elevated serotonin concentrations increased expression of the serotonin degradation gene monoamine oxidase-A in whole blood.
• Increased circulating serotonin upregulated expression of serotonin receptor 7 in whole blood.
• Infusion of 5-hydroxy-l-tryptophan (5-HTP) elicits rapid, acute changes in whole-blood gene expression relative to control after 3 d of treatment.

## Graphical Abstract

**Figure:**
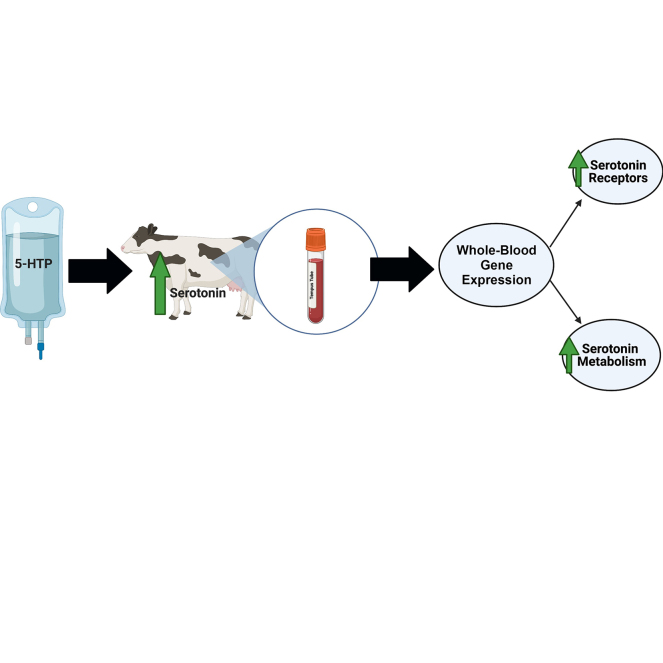
**Summary:** It has been established that serotonin plays a multifunctional role within the body, with recent research demonstrating serotonin's ability to alter whole-blood gene expression and immune function in dairy calves. However, the ability of serotonin to modulate whole-blood gene expression in the adult dairy cow is unknown. The current study demonstrated that elevation in circulating serotonin alters whole-blood expression of serotonin receptor, transport, and degradation genes. Further, these data indicate the potential effect of serotonergic manipulation on whole-blood serotonin-related and immune-related genes in the adult dairy cow.

Serotonin was originally discovered due to its role as a vasoconstrictor, but recent research has focused on its role as a hormone and biogenic amine (Rapport et al., 1948; Mohammad-Zadeh et al., 2008). This dynamic monoamine plays many critical roles in physiology, with peripheral serotonin acting to modulate mammary physiology, immunoregulatory properties, endocrine function, and gastrointestinal health (Berger et al., 2009; Herr et al., 2017). Serotonin is derived from the amino acid l-tryptophan in a 2-step enzymatic process. The rate-limiting step of serotonin synthesis is catalyzed by the enzyme tryptophan hydroxylase (**TPH**), which converts l-tryptophan to 5-hydroxy-l-tryptophan (**5-HTP**). Upon synthesis of 5-HTP, decarboxylation occurs via aromatic amino acid decarboxylase to produce serotonin. Serotonin can then be inactivated by monoamine oxidase (**MAO**), with the primary inactivation occurring via MAO-A (Mohammad-Zadeh et al., 2008). Two pools of serotonin—a peripheral pool and a neuronal pool—reside within the body, and each pool is considered distinctly separate. Thus, serotonin synthesized and secreted in the periphery resides in the periphery (Walther and Bader, 2003). Interestingly, although one of serotonin's classical functions is as a neurotransmitter, approximately 95% of serotonin is actually found in the periphery (Mohammad-Zadeh et al., 2008). Lately, interest has been focused on understanding peripheral serotonin's pertinence in physiology. To appreciate serotonin's regulatory roles in the periphery in various systems, understanding serotonin's metabolic and signaling pathways is critical.

Serotonin acts via 14 different cell-surface receptors, which are classified into 7 families and expressed ubiquitously. The majority of the serotonin subtype receptors are G-protein coupled receptors, with the type 3 serotonin receptors being ligand-gated ion channels (Hannon and Hoyer, 2008). Serotonin action has been demonstrated to be mediated by different subtypes of the 7 serotonin mammalian receptor classes as well as the serotonin reuptake transporter (**SERT**) and serotonylation, which is a receptor-independent signaling mechanism that proceeds from serotonin being covalently bound to cellular proteins (Hannon and Hoyer, 2008; Berger et al., 2009). Interestingly, a variety of immune cells have been demonstrated to be able to reuptake serotonin via SERT and then metabolize serotonin via MAO. Monocytes, macrophages, and T-cells express TPH1, indicating their ability to synthesize serotonin, thus supporting serotonin's function as an immunomodulatory amine (Herr et al., 2017). Research has demonstrated that a unique serotonin–immunity relationship exists, with serotonin action inhibiting tumor necrosis factor-α (**TNF-α**) production (Arzt et al., 1991; Maes, 2001; Idzko et al., 2004; Kubera et al., 2005). Further, dendritic cells secrete a variety of cytokines, with serotonin modulating dendritic cell cytokine secretion in a maturation-dependent manner (Idzko et al., 2004). Recently, serotonin receptors were found to be expressed on preweaned bovine immune cells, with peripheral leukocytes containing all of the machinery to synthesize (TPH1), reuptake and transport (SERT), and degrade (MAO) serotonin (Marrero et al., 2020). Although serotonin receptor dynamics have been researched for decades in humans and rodents, the presence and activity in bovine blood and leukocytes have yet to be fully understood.

Manipulation of the serotonergic axis in preweaned calves through oral administration of fluoxetine (a selective serotonin reuptake inhibitor) and 5-HTP elicited changes in multiple serotonin receptor subtypes, serotonin metabolism-related genes, and immune-related genes in peripheral leukocytes (Marrero et al., 2020). Further, 5-HTP supplementation in colostrum and milk fed to calves increased mRNA abundance of genes related to both the innate and adaptive immune systems (Hernández-Castellano et al., 2018). However, serotonin receptor dynamics and metabolism in whole blood of lactating dairy cows has yet to be examined in response to serotonergic manipulation. Therefore, our objective was to explore alterations in the expression of key serotonin receptor and metabolism genes and immune-related genes in whole blood of lactating dairy cows in response to 3 d of 5-HTP infusion. We hypothesized that 5-HTP infusion would modulate serotonin signaling and metabolism genes in whole blood in response to 5-HTP infusion.

All experimental procedures were approved by the College of Agriculture and Life Sciences Animal Care and Use Committee at the University of Wisconsin–Madison and were strictly followed (A005903). More extensive methodology and information are provided in Connelly et al. (2021). Briefly, cows were housed in an enclosed tiestall barn at the Dairy Cattle Center at the University of Wisconsin–Madison, milked twice daily, and fed the standard herd lactating-cow diet formulated to meet or exceed lactational requirements. Twelve lactating (212.17 ± 20.04 DIM; 2.5 ± 0.26 average lactation parity, 41.21 ± 2.31 kg/d average milk yield) multiparous Holstein dairy cows were blocked by parity, milk production, and DIM in a randomized complete block design and randomly assigned to intravenous infusion of either 1 L of 1.5 mg/kg 5-HTP dissolved in saline (n = 6) or 1 L of saline solution (control; n = 6) for 3 consecutive days. The dose and duration of 5-HTP were based on previous research in our laboratory (Laporta et al., 2015). Intravenous infusion of 5-HTP (H9772, Sigma-Aldrich) was calculated (on a mg/kg of BW basis) for each cow before treatment and was mixed in saline until fully dissolved in solution. The fully dissolved solution was then sterilized by filtration, labeled, and stored at 4°C until use. Body weights were recorded the day before the start of the experimental period, at which time a single catheter was inserted into the jugular vein. Infusions were administered at approximately 0800 h via jugular catheter at a constant rate over 1-h periods on the first (d 0), second (d 1), and third (d 2) days of infusion.

Baseline blood samples were collected 1 d before administration of treatment. Before blood collection from the catheter, 8 mL of whole blood was drawn and discarded to remove any residual heparinized saline. Upon termination of the third and final infusion, additional blood samples were collected immediately (48 h relative to termination of the first infusion), 8 h later (56 h relative to termination of the first infusion), and 24 h later (72 h relative to termination of the first infusion; Figure 1A). Blood samples were collected in 10-mL Vacutainer serum tubes (367820; BD), allowed to clot at room temperature, and then isolated by centrifugation at 3,000 × *g* for 20 min at 4°C for serum isolation. Isolated serum was then aliquoted and stored at −80°C. For isolation of total RNA from whole blood, 3 mL of blood was collected via the coccygeal vein into Tempus blood RNA tubes (Applied Biosystems) containing 5 mL of RNA stabilization solution and vigorously shaken for 20 s immediately upon collection. After collection, the Tempus tubes were stored at −80°C until analysis. Concentrations of TNF-α were attempted in serum using a bovine ELISA (EBTNF, Invitrogen) but fell below the assay detection limit and thus were not reported. Concentrations of IL-8 were measured in serum using a human IL-8 ELISA (D8000C, R&D Systems Inc.) as previously described (Zinicola et al., 2019). The intra- and interassay coefficients of variation for serum IL-8 were 4.56 and 7.02%, respectively. Total RNA was extracted from whole blood using the Tempus Spin RNA Isolation Kit (Applied Biosystems) per the manufacturer's instructions as previously described (Carvalho et al., 2017). Total RNA concentration was determined by quantification of absorbance ratios by Nanodrop spectrophotometer (ND-1000, Nanodrop Technologies). One microgram of total RNA was then reverse transcribed to synthesize cDNA using a High Capacity cDNA Reverse Transcription Kit (4368814, Applied Biosystems). After reverse transcription, cDNA was diluted (1:5) in molecular-grade water. Quantitative real-time PCR was performed using a CFX96 Touch Real-Time PCR Detection System (Bio-Rad Laboratories). Expression of genes involved in serotonin signaling and metabolism as well as immune-related genes in whole blood samples were evaluated with primer sequences given in Table 1. Reaction mixtures, cycling conditions, and primer design were performed as previously described (Laporta et al., 2013). The geometric mean of cyclophilin A and β-actin was used for standardization across samples. Baseline mRNA expression was not different between treatments. Data were analyzed using the 2^−ΔΔCt^ method, with saline-infused cows at 48 h serving as the internal control (Livak and Schmittgen, 2001).

**Figure 1:**
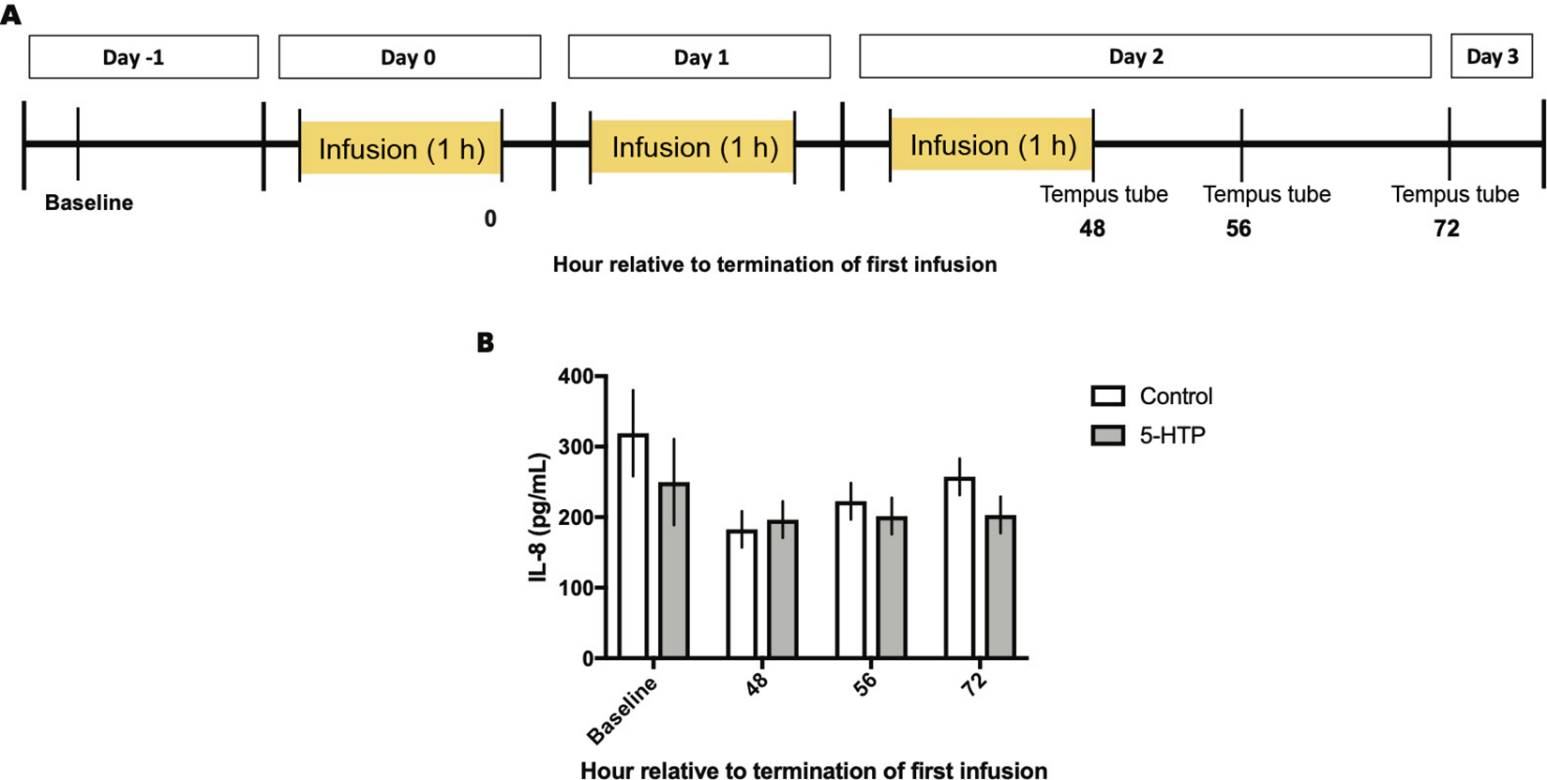
Schematic representation of experimental design and sampling timeline. (A) Twelve lactating multiparous Holstein dairy cows were blocked by parity, milk production, and DIM in a randomized complete block design and randomly assigned to intravenous infusion of either 1 L of 1.5 mg/kg 5-hydroxy-l-tryptophan (5-HTP) dissolved in saline (n = 6) or 1 L of saline solution (control; n = 6) for 3 consecutive days. (B) Serum IL-8 concentrations in multiparous Holstein dairy cows receiving 1 L of 1.5 mg/kg 5-HTP dissolved in saline or 1 L of saline solution for 3 consecutive days. Data are presented as LSM ± SEM.

**Table 1:** Primer sequences used for quantitative real-time PCR performed in lactating multiparous Holstein dairy cows^1^

Gene^2^	Forward (5′–3′)	Reverse (3′–5′)
*TPH1*	AGAGAATTTACCAAGACAATCAAG	CTTAGCAAGGGCATCACTGAC
*MAOA*	CATCGATAACTGCCCTGTGG	ATTGCACGGCTGTTCTATGG
*SERT*	GAAGCTGTTGGAGGAGTTCG	CCAGCAGATCTTCCAGAACC
*HTR2A*	TCCTGTTTGTGGTGATGTGG	GGTTGACTGCTGAGGAGAGG
*HTR2B*	CTGGCTTCCTTCTTCACACC	AACCATGTTAGGCGTTGAGG
*HTR4*	ATGGACAAACTTGATGCTAATGTG	TCACCAGCACCGAAACCAGCA
*HTR7*	AATCATTTGCCGAGACTTCG	CGGATCCACAGAAAACAAGG
*IL8*	ATTCCACACCTTTCCACCCC	CCTTGGGGTTTAGGCAGACC
*TNFA*	CCAGACCAAGGTCAACATCC	CGGCATAGTCCAGGTAGTCC
*ACTB*	ACTTGCGCAGAAAACGAGAT	CACCTTCACCGTTCCAGTTT
*PPIA*	CACCGTGTTCTTCGACATCG	ACAGCTCAAAAGAGACGCGG

1 All primers were designed and sequences were obtained as in Laporta et al. (2013). Reactions were run at an annealing temperature of 60°C. The geometric mean of *ACTB* and *PPIA* was used as the housekeeping parameter.

2 *TPH1* = tryptophan hydroxylase 1; *HTR2A* = serotonin receptor 2a; *HTR2B* = serotonin receptor 2b; *HTR4* = serotonin receptor 4; *HTR7* = serotonin receptor 7; *MAOA* = monoamine oxidase A; *SERT* = serotonin reuptake transporter; *IL8* = IL-8; *TNFA* = tumor necrosis factor α; *ACTB* = β-actin; *PPIA* = cyclophilin A.

Data were analyzed using the MIXED procedure of SAS (version 9.4, SAS Institute Inc.). Fixed terms in the model for serum IL-8 concentrations included treatment, block, covariate, time, and treatment × time. Covariate values were a single baseline blood sample taken before treatment administration. Fixed terms in the model for gene expression included treatment, block, time, and treatment × time. Hour was considered the repeated measure, and to account for autocorrelated errors the spatial power structure was used within the SAS MIXED procedure. The random statement in all models included cow (block). Normality of residuals was examined for each variable, and responses that violated the assumptions of normality were then transformed. Transformations were based on diagnostic plots and overall model fit, with either rank or log transformation then performed on response variables to improve normality of residuals (*SERT*, *HTR4*, and *HTR7*). Pairwise comparisons were made between groups within each time point with the adjustment of Tukey to account for multiple comparisons. Data are presented as least squares means ± standard error of the mean. Statistical significance was declared at *P* ≤ 0.05.

We previously reported that administration of 5-HTP increased circulating serotonin concentrations by ~800 ng/mL across the experimental period in cows infused with 5-HTP (Connelly et al., 2021). Circulating IL-8 concentrations were unchanged in response to 5-HTP infusion across the experimental period (*P* > 0.05; Figure 1B). Circulating concentrations of TNF-α were analyzed but fell below the detection limits of the assay and thus not reported. Cows infused with 5-HTP had increased whole-blood expression of *MAOA* across the experimental period (*P* = 0.01), with mirrored increases in *SERT* and *MAOA* 48 h after termination of the first infusion (*P* = 0.05 and *P* = 0.009, respectively; Figure 2A and B). However, no differences between 5-HTP and control cows were observed in either gene at 56 and 72 h after termination of first infusion. Further, *MAOA* expression exhibited a time (*P* = 0.005) and treatment × time (*P* = 0.02) interaction. Expression of *TPH1*, the enzyme that catalyzes the synthesis of 5-HTP from l-tryptophan, was unaltered across the experimental period, as was expression of *HTR2A* and *HTR2B* (*P* > 0.05; Figure 2C–E). However, other serotonin receptors (*HTR4* and *HTR7*) exhibited dynamic expression across the experimental period. Expression of *HTR4* mRNA tended to be upregulated 24 h after termination of the third and final infusion in cows treated with 5-HTP relative to the control (*P* = 0.10; Figure 2F). Relative mRNA expression of *HTR7* was increased (*P* = 0.002) across the experimental period in cows infused with 5-HTP relative to the control. Relative expression of *HTR7* mRNA was upregulated immediately and 24 h after termination of the third and final infusion in cows treated with 5-HTP relative to the control (48 h, *P* = 0.005; 72 h, *P* = 0.06; Figure 2G). No treatment differences were observed in *IL8* mRNA expression (Figure 2H) or *TNFA* expression (Figure 2I) in whole blood. Interestingly, whole-blood expression of *TNFA* was decreased (*P* = 0.14) in cows infused with 5-HTP relative to the control at 48 h after termination of first infusion, but no differences between treatments were detected at 56 and 72 h after termination of first infusion.

**Figure 2:**
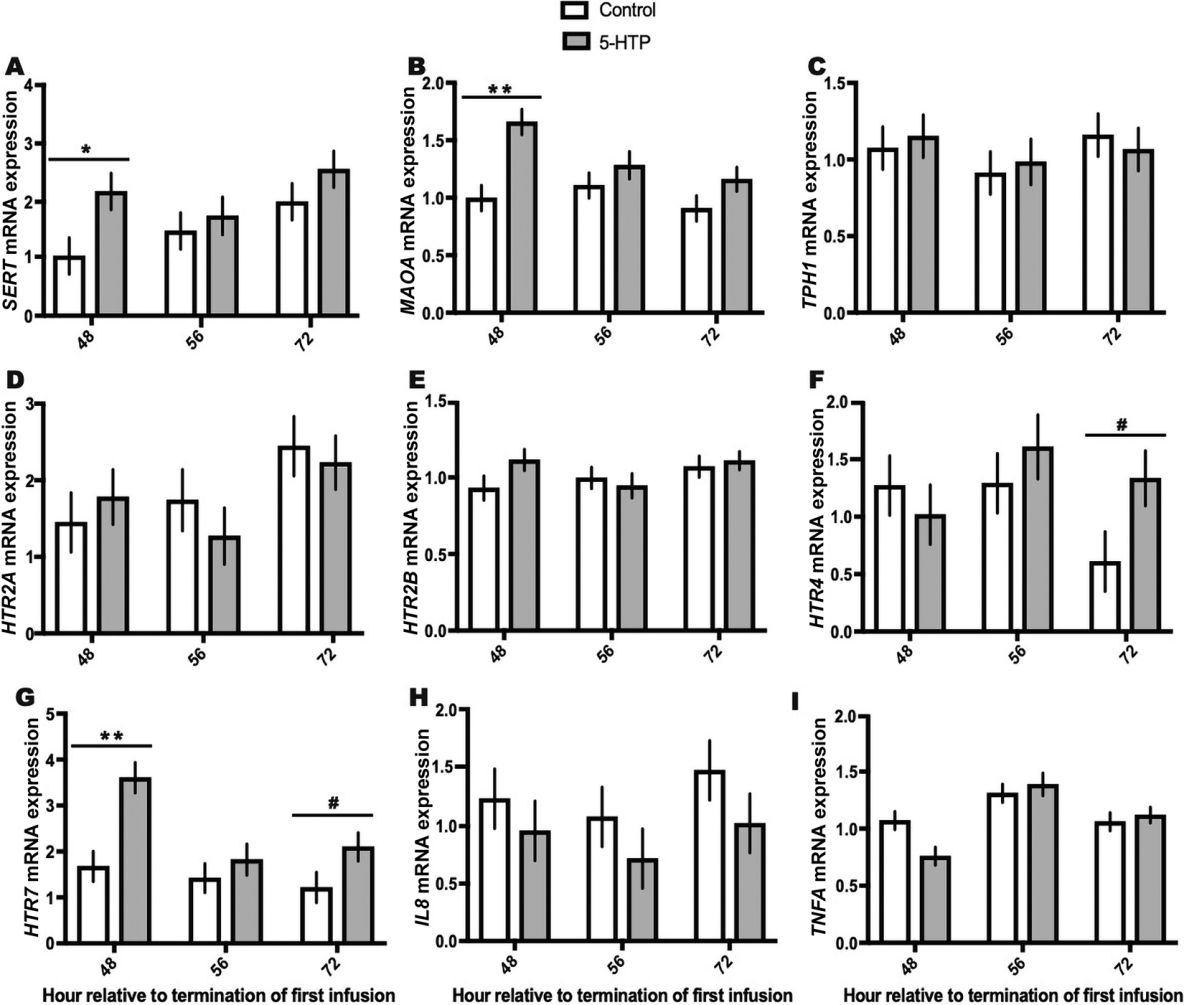
Whole-blood mRNA expression of (A) *SERT*, (B) *MAOA*, (C) *TPH1*, (D) *HTR2A*, (E) *HTR2B*, (F) *HTR4*, (G) *HTR7*, (H) *IL8*, and (I) *TNFA* in multiparous Holstein dairy cows receiving either 1 L of 1.5 mg/kg 5-hydroxy-l-tryptophan (5-HTP) dissolved in saline or 1 L of saline solution (control) for 3 consecutive days. Data are presented as LSM ± SEM. *SERT* mRNA: time (*P* = 0.0002) and treatment × time (*P* = 0.007). *MAOA* mRNA: treatment (*P* = 0.01), time (*P* = 0.005), and treatment × time (*P* = 0.02). *HTR2A* mRNA: time (*P* = 0.002). *HTR4* mRNA: time (*P* = 0.07) and treatment × time (*P* = 0.05). *HTR7* mRNA: treatment (*P* = 0.002). *IL8* mRNA: time (*P* = 0.003). *TNFA* mRNA: time (*P* = 0.0009) and treatment × time (*P* = 0.06). Pairwise comparisons were made between groups within each time point using differences of LSM with the adjustment of Tukey. Symbols denote statistical significance between groups at each time point: **P < 0.01; *P < 0.05; #0.10 < *P* > 0.05.

Research has focused on manipulation of the serotonergic axis in preweaned calves via 5-HTP and fluoxetine administration, which demonstrated that increased circulating serotonin concentrations have immunomodulatory effects and elicit alterations in mRNA of serotonin-related genes (Hernández-Castellano et al., 2018; Marrero et al., 2020). However, serotonin receptor dynamics and metabolism genes in whole blood of lactating dairy cows have yet to be explored in response to 5-HTP administration. Previous research evaluating serotonin's impact on calcium metabolism in the late-lactation dairy cow demonstrated successful manipulation of serotonin after 2 doses of 5-HTP. Thus, we sought to replicate similar dosing schematics and focus on the time period after 3 d of dosing to ensure that serotonin concentrations in blood were altered. These results support the published findings of 5-HTP administration altering expression of serotonin-related genes in the preweaning calf (Marrero et al., 2020) and, to our knowledge, is the first data set to report that administration of 5-HTP elicits alterations in expression of whole-blood serotonin metabolism genes and receptors in the lactating dairy cow.

Previous work conducted in preweaned dairy calves demonstrated that elevated circulating serotonin concentrations via 5-HTP administration modulate expression of serotonin signaling and metabolism genes as well as immune-related genes (Hernández-Castellano et al., 2018; Marrero et al., 2020). However, the dairy calf is born with a naïve immune system, and its adaptive immunity develops in the ensuing months after birth (Clover and Zarkower, 1980; Kampen et al., 2006). Thus, the adult lactating dairy cow has a very different physiological system when considering immunomodulation and blood mRNA expression.

No changes in expression of *TPH1* were observed herein. However, lack of *TPH1* expression change is not surprising because *TPH1* is the enzyme that catalyzes the conversion of tryptophan to 5-HTP, which is bypassed when administering the precursor (5-HTP) to serotonin. These data in the adult dairy cow are congruent with peripheral leukocyte expression changes in the preweaned calf in response to 5-HTP administration in Marrero et al. (2020). Increased circulating serotonin concentrations did induce alterations in mRNA expression of serotonin transport and degradation genes in the lactating dairy cow. Two key serotonin-related genes, *SERT* and *MAOA*, were upregulated immediately after termination of the third and final infusion (48 h after termination of first infusion). Increases in *SERT* mRNA would indicate increased serotonin uptake in cows infused with 5-HTP, with a corresponding inactivation of serotonin through increased *MAOA* expression. Serotonin is believed to be rapidly metabolized by MAO (Mohammad-Zadeh et al., 2008), which is supported in this study by robustly increased *MAOA* expression immediately after termination of the final infusion (48 h) and congruent with *MAOA* expression in mammary tissues in response to 5-HTP infusion (Connelly et al., 2021). However, the role of SERT is not just reuptake of serotonin for potential degradation. Expression of SERT also mediates serotonin signaling and facilitates accumulation and storage of serotonin within cells that cannot synthesize serotonin independently (Horseman and Collier, 2014).

Research has shown that monocytes, macrophages, and lymphocytes all express SERT and monocytes, macrophages, dendritic cells, and T cells are able to degrade serotonin due to the presence of MAO-A (Herr et al., 2017). Although this study did not quantify immune cell populations, no differences in white blood cell populations were noted in the 10-d 5-HTP supplementation period in preweaned dairy calves (Marrero et al., 2020). Though the immune systems of the adult dairy cow and preweaned calf are substantially different, these data reiterate that increased supply of the serotonin precursor 5-HTP elicits changes in mRNA of whole-blood serotonin metabolism and transport genes in response to 5-HTP infusion in the lactating dairy cow.

Elevation of circulating serotonin not only altered serotonin metabolism and transport genes in whole blood but also induced an increase in *HTR7* mRNA expression in cows infused with 5-HTP. We observed increased expression of *HTR7* in cows treated with 5-HTP and a tendency for increased expression of *HTR4* 24 h after termination of the third and final infusion (72 h) relative to control cows. In comparison, Marrero et al. (2020) observed increased *5HTR4* expression in response to 5-HTP treatment but no changes in *HTR7* expression. Serotonin receptor 7 has been shown to be predominantly expressed on naïve T cells, with enhanced T-cell activation occurring via serotonin stimulation (León-Ponte et al., 2007). Further, both serotonin receptor 4 and serotonin receptor 7 have been shown to mediate the release and secretion of IL-8 and TNF-α in dendritic cells (Idzko et al., 2004). Expression of *HTR2A* was unchanged herein and in the study by Marrero et al. (2020). Additionally, no changes were observed in *HTR2B* mRNA expression in whole blood in response to 5-HTP, opposing previous research in the preweaned bovine (Marrero et al., 2020). However, it is important to note that the immune systems of calves and adult dairy cows are markedly different and thus could lead to differing responses and stimulation of serotonergic immune signaling. A further consideration is that different dosing lengths and rates of 5-HTP were used in each study.

Cows infused with 5-HTP exhibited numerically lower circulating IL-8 concentrations and decreased whole-blood expression of *IL8*; however, no differences due to treatment were observed. Circulating TNF-α concentration values were below detection limit of the assay, and when mRNA expression was analyzed in whole blood, *TNFA* expression was numerically decreased in 5-HTP-infused cows 48 h after termination of the first infusion relative to control cows. Although no significant *TNFA* changes occurred in this study, early work demonstrated that serotonin inhibited TNF-α synthesis from LPS-stimulated human mononuclear cells (Arzt et al., 1991). This serotonin–TNF-α link was supported by data that demonstrated that dendritic cells exhibited serotonin-mediated inhibition of TNF-α production due to HTR4 and HTR7 activation (Idzko et al., 2004). Further, in the current study, *HTR7* expression was upregulated in 5-HTP-infused cows immediately after termination of the third and final infusion, in tandem with a numerical decrease in *TNFA* expression in 5-HTP-infused cows at the same time point (48 h). However, research in the preweaned calf supplemented with 5-HTP showed no alterations in *TNFA* expression (Hernández-Castellano et al., 2018; Marrero et al., 2020).

Although this study focused on whole-blood transcripts, TNF-α responses in the mammary gland have been shown to be dynamic across lactation, with high-yielding late-lactation cows having lower abundance of TNF-α transcripts in isolated milk leukocytes relative to early- and mid-lactation time points (Mukherjee et al., 2013). The periparturient cow also tends to have higher concentrations of circulating TNF-α (Kasimanickam et al., 2013) due to systemic inflammation in response to the events surrounding parturition and the adaptation to lactation (Bradford et al., 2015), which collectively results in increased circulating TNF-α concentrations in early lactation. Thus, the lack of measurable changes in circulating TNF-α may be due to the late stage of lactation of cows in the current study.

Collectively, these data support the need for further research in understanding serotonin's potential immunomodulatory effects via 5-HTP administration in the lactating dairy cow. Accumulating evidence suggests a basic immunoregulatory function of serotonin in the immune system, with the data herein showing that elevated serotonin modulates serotonin transport, metabolism, and receptor dynamics in whole blood in lactating dairy cows. Further research is needed to study how altering the serotonergic axis may modulate immune regulation during steady-state physiology, immune challenges, inflammatory states, and various stages of lactation. Although these data suggest a potential role for serotonin beyond the current improvement in calcium metabolism, an understanding of this complex molecule and the roles it may play in the mature cow as she progresses through gestation and into lactation is much needed.
